# Reproductive performance and milk yield of rabbits fed diets supplemented with garden cress (*Lepidium sativum*) seed

**DOI:** 10.1038/s41598-022-21449-0

**Published:** 2022-10-12

**Authors:** Yassmine M. El-Gindy, Soliman M. Zahran, Mohamed H. Ahmed, Azza Y. Idres, Safaa H. Aboolo, Sabrin A. Morshedy

**Affiliations:** 1 Fish and Animal Production Department, Faculty of Agriculture (Saba Basha), Alexandria University, P.O. Box 21531, Alexandria, Egypt; 2 Animal Production Department, Faculty of Agriculture, Omar Al-Mukhtar University, Elbea, Libya; 3 Pathology Department, Animal Health Research Institute (AHRI), Agriculture Research Center (ARC), P.O. Box 12618, Giza, Egypt

**Keywords:** Immunology, Physiology

## Abstract

Recently, phytochemicals in plants have evoked interest as sources of naturally beneficial substances and as alternatives to antimicrobials. Based on these benefits, it was hypothesized that garden cress (GC; *Lepidium sativum*) supplementation could overcome the negative impacts of severe heat stress on the reproductive and lactation performance, physiological parameters, and antioxidant status of rabbits. Twenty-four mature V-line does (6 months old) with an average body weight of 3.65 ± 0.54 kg were randomly assigned into four homogenously equal groups (n = 6) according to the level of supplemented GC seeds. Rabbits in the control group were fed a basal diet without GC seeds (GC 0), the other three treatment groups (GC 3, GC 4.5, and GC 6) were fed a basal diet supplemented with GC seeds at levels 3, 4.5 and 6%, respectively. Investigations revealed that the inclusion of 3% GC enhanced the litter weight of heat-stressed rabbits on the 7th, 14th, and 21st days. Furthermore, treatment with 3% and 6% GC seeds increased the milk yield on the 28th day. the most of lipid profile parameters, such as cholesterol, triglyceride, high-density lipoprotein (HDL), low-density lipoprotein (LDL), serum urea levels, and antioxidant status improved in rabbits supplemented with 6% GC. In conclusion, the dietary supplemention of GC seed at 6% increased milk yield at 28^th^ day “weaning age”, consequently, improved the blood lipid profile and antioxidant status. Further studies should be conducted to commercialize theusage of garden cress seeds as a supplement in rabbits.

## Introduction

Medicinal plants have gained significant attention for being used as animal feed additives to produce quality animals that the customers worldwide could accept, cover essential nutrient needs, and improve the rabbit's health and reproductive performance^1^. Medicinal plants used as supplements can directly influence milk yield and composition and the number of kits born alive. Therefore, the kits' survival and growth during lactation are enhanced^2^. Additionally, medicinal plants fed to rabbits can affect the immune status of their kits at weaning^3^ by increased production and storage of immune system cells in the spleen^4^..These plants are a prolific source of secondary metabolites, with important functions both in vivo and in vitro during ovarian folliculogenesis and steroidogenesis in many animal species^5^. Hence, with technological development, there is an increased implication of these substances in assisted reproductive technology^5^.

Garden cress (GC; *Lepidium sativum L.*) is a fast-growing annual herb, belonging to the Brassicaceae family. It is native to Egypt and the west of Asia and it is presently being cultivated worldwide^6^. Furthermore, GC can grow under any climatic and soil conditions^5^. Morphologically, GC seeds are brownish-red and oval-shaped, with their seed coat accounting for 12–17% and the embryo accounting for 2–3% of the seeds. It has been reported that GC is used for different medicinal applications^7^**.** Specifically, although its seeds are bitter, they have health-promoting and antioxidant properties, including thermogenic, depurative, rubefacient, galactagogue, aphrodisiac, ophthalmic, antiscorbutic, antihistaminic, diuretic and carminative effects [8, 9]. GC seeds can also be nephroprotective agents^10^. Additionally, GC seeds possess antibacterial and antifungal properties^9^ and can be used to treat asthma, coughs with expectoration, sprains, leprosy, skin disease, dysentery, diarrhea, splenomegaly, dyspepsia, lumbago, leucorrhea, scurvy, and seminal weakness^11^.

GC seeds have also been reported to possess a significant number of total phenolic compounds (approximately 36.41 mg gallic acid equivalents /100 g), with high levels of α, δ, and ϒ-tocopherols^8^. furthermore GC seed contain a high content of essential fatty acids ( 30.6% oleic; and 29.3% linolenic) and rich lignin/antioxidant concentrations, which can help stabilize n-3 polyunsaturated fatty acids^12^. The active ingredients in GC seeds are displayed in Table 1 according to Halaby et al.^10^. Therefore, based on its beneficial properties, this study hypothesizes that if used as a feed additive at different levels in rabbit diets, GC seeds could improve the reproductive performance, milk yield, and liver and kidney function, including the antioxidant status of V-Line does.

**Table 1:** Active components of garden cress seeds (per dry matter).

Active components	Amount
Total antioxidant activity DPPH (μg/mL)	96.64
Tannins (mg/100 g)	13.95
Vitamin A (μ/100 g)	90.00
Vitamin E (ppm)	258.74
Vitamin C (mg/100 g)	10.62
Thiamine (mg/100 g)	00.59
Riboflavin (mg/100 g)	00.61
Niacin (mg/100 g)	14.30
total saturated fatty acid (g/100 g)	16.71
monounsaturated (g/100 g)	42.61
polyunsaturated fatty acids (g/100 g)	40.68
**Flavonoids (ppm) in garden cress seeds**	
Kampferol	709.66
Narengin	610.55
Querciterin	123.73
Apignin	96.34
Narenginin	54.42
**Polyphenolic compounds (ppm) in garden cress seeds**	
Benzoic acid	186.94
Pyrogallol	137.51
Epicatechen	135.97
Protocatechuic acid	102.14
Salicylic acid	91.50
Catechol	65.64
Catechein	16.52
Vanillic acid	43.34
Caffiene	46.22

Source: Halaby et al.^9^.

## Materials and methods

### GC source and preparation

GC seeds were obtained from a local market in Bakos, Alexandria, Egypt, during the harvest season between 2019 and 2020. These seeds were washed under running water and dried in a drying oven (temperature under 40 °C) for 3 days, after which the dried seeds were ground to a fine powder using an electric grinder (500 g, DAMAI, Zhejiang, China). Finally, the ground seeds were stored in an airtight container until mixing and pelleting for the experimental diets. The diets were pelleted to a diameter of 6 mm. All GC seeds were purchased, managed, and the research was conducted in compliance with relevant institutional, national, and international guidelines and legislation.

### Animals, animal management, and experimental design

The experiment was conducted at a rabbit’s laboratory in the Faculty of Agriculture, Saba Basha, Alexandria University, Egypt. Twenty-four mature V-line doe rabbits, previously defined^13^, (6 months old) with an average body weight of 3.65 ± 0.54 kg were used in the study. Does were randomly allocated into four homogenously equal groups (n = 6) according to the level of supplemented GC seeds. Rabbits in the control group were fed a basal diet without GC seeds (GC 0), those in the other three treatment groups (GC 3, GC 4.5, and GC 6) were fed a basal diet supplemented with GC seeds at levels 3, 4.5 and 6%, respectively. The experiment lasted for four months (March–June), including one month (before mating) as an adaptation protocol. The basal pellet diet comprised 16% corn, 7% barley, 19% wheat bran, 20% soybean meal, 44% protein, 24% alfalfa hay, 2% beet molasses, 10% wheat straw, 0.20% calcium carbonate, 0.80% dicalcium phosphate, 0.50% sodium chloride, 0.15% l-lysine, 0.05% methionine, and 0.3% of the premix.The rabbits were individually housed in naturally ventilated and galvanized wire-caged batteries with sufficient lighting. In GC experimental diets, the corn and soybean meal was replaced with GC seeds at levels 3, 4.5, and 6%. Nevertheless, all diets covered the daily nutritional requirements of the rabbits (17.2% protein and 2453.40 kcal/kg diet digestible energy), as reported by NRC^14^. Diets and clean tap water were supplied ad libitum throughout the experimental period. The study did not use any of methods of anesthesia or rabbit euthanasia. The present study protocol was reviewed and approved by the Ethical Committee of Alexandria University [Approval No. AU: 14/19/01/16/2/6]. All authors in this study complied with the ARRIVE guidelines and confirmed all experiments were performed in accordance with the relevant guidelines and regulations.

### Reproductive performance and milk yield

After a month adaptation period, the does were introduced to untreated bucks in their cages for mating. They were also weighed at mating, palpation, and parturition. Their conception rates were tested on the 15th-day post-coitus by abdominal palpation. Twenty-six days after mating, nest boxes were prepared with rice straw, after which their gestation periods and pre-weaning mortality were determined. Litter size, litter weight, and bunny weight were also recorded at birth, on the 7th, 14th, and 21st days post partum, and then at weaning (28th day of age). Finally, milk yield was measured during the four weeks of lactation by weighing the doe immediately before and after suckling.

### Haematological parameters and blood biochemicals

The does were prevented from eating for some hours before blood sampling at weaning. Subsequently, while 7 mL of blood samples were collected individually using sterile disposable needles from marginal ear veins, approximately 2 mL of blood was put into a test tube containing ethylenediaminetetraacetic acid as an anticoagulant for haematological analyses. The erythrocytes and leukocytes were manually counted using a standard Neubauer cell counting chamber. Specifically, while the erythrocytes were counted after diluting blood samples 200 times with a diluting fluid (10% of sodium sulfate, 2% of sodium chloride, and mercuric chloride 1% solution), leukocytes were counted after diluting blood samples 20 times with a diluting fluid (1.5% of glacial acetic acid solution and a few crystals of gentian violet). Next, packed cell volume was manually measured after centrifugation at 750×*g* for 20 min. In contrast, hemoglobin concentration (Hb) was determined calorimetrically using commercial kits (Biodiagnostic Co., Cairo, Egypt) according to a previous study^15^, after which platelets were finally counted according to another study^16^. Subsequent calculations of the mean cell volume (MCV), mean cell haemoglobin (MCH), and mean cell haemoglobin concentration (MCHC) were also conducted^17^.

The remaining 5 mL of blood sample was left at room temperature to coagulate. The serum was separated by centrifugation for 15 min (at 70×*g*) to obtain clean supernatant serum and stored in vials at − 20 °C for later analyses. Subsequently, frozen serum was thawed and assayed calorimetrically for total lipid, cholesterol, triglyceride (TG), and high-density lipoprotein (HDL) concentrationsusing commercial kits produced by Biodiagnostic Co. Cairo, Egypt. Very-low-density lipoprotein (vLDL) was calculated by dividing the values of TG by a factor of five according to a study^18^. Low-density lipoprotein (LDL) concentration was calculated using the following formula:

$${\text{LDL }} = {\text{ cholesterol }}{-} \, \left( {{\text{HDL }} + {\text{ vLDL}}} \right).$$

The levels of serum urea-N and creatinine, and the activities of serum glutamic-oxaloacetic transaminase (GOT) and glutamic pyruvic transaminase (GPT) were also determined by commercial kits produced by Biodiagnostic Co., Cairo, Egypt. Total antioxidant capacity (TAC) was measured following the manufacturer’s recommendations (Biodiagnostic, Egypt). superoxide dismutase (SOD) was determined according to the methods of^17^.

### Statistical analysis

Results obtained from the experiment were subjected to a one-way analysis of variance (ANOVA) using SPSS for Windows (v.16.0., SPSS Inc., Chicago, IL, USA). Before the statistical analysis, percentage values were transformed into Arc–Sin values to approximate the normal distribution. Then, significant differences (*P* = 0.05) between the means were separated using the Duncan’s Multiple Range Test option of the same software, using the following statistical model:

$${\text{One - way model: Y}}_{{{\text{iK}}}} = \, \mu + {\text{ X}}_{{\text{i}}} + {\text{ e}}_{{{\text{ik}}}}$$

where Y_iK_ = the response variable; µ = the overall mean; X_i_ = fixed treatment effects (0%, 3%, 4.5%, and 6% of GC seeds); and e_ik_ = the residual error.

### Ethics approval and consent to participate

All female rabbits’ handling procedures at different physiological stages followed the instructions and guidelines of the Experimental Animal Ethics Committee, Alexandria University, Egypt (Ethics Consent Approval No AU: 14/19/01/16/2/6) which adhered to the ARRIVE guidelines.

## Results

### Body weight and reproductive performance

The body weights of the experimental rabbits throughout mating, palpation, and parturition did not differ significantly due to the dietary supplementation of GC seeds (Table 2). Furthermore, dietary supplementation of GC seeds did not affect the gestation periods and conception rates (*P* > 0.05). Nevertheless, while the GC 3% group displayed the most outstanding value of conception rate, that of the CG 0% and 4.5% groups were numerically the lowest.

**Table 2:** Effect of dietary levels of Garden grass levels on female rabbit body weight and reproductive parameters of female’s rabbits.

Parameters	Garden cress seeds (%)				SEM	P value			
	0	3	4.5	6		Treat	Linear	Quadratic	Cubic
**Doe weight (kg) at:**									
Mating	3.66	3.63	3.60	3.65	54.35	0.986	0.937	0.847	0.847
Palpation	3.89	3.88	3.89	3.90	53.26	1.000	0.996	0.996	0.996
Parturition	3.65	3.62	3.58	3.64	54.00	0.980	0.919	0.821	0.821
**Reproductive performance**									
Conception rate (0–1)	0.67	1.00	0.67	0.83	0.09	0.472	0.829	0.631	0.142
Gestation period, day	29.75	29.83	29.50	30.00	0.15	0.730	0.565	0.372	0.372

*SEM* stander error means.

Table 3 summarizes the litter size and litter weight data. Results showed that both the GC 3% and 6% groups had significantly (*P* < 0.01) improved litter sizes at birth and on the 14th day. Litter weight of GC 3% group on the 14th day was also significantly improved compared with that of the control and GC 4.5% rabbits. Although the GC 3% group recorded the largest litter size at birth, 7th and 14th (*P* < 0.05) and litter weight on the 14th and 21st day as compared with the other groups. Besides, there was no significant difference in litter size between all experimental groups on the 21st day, at weaning and pre-weaning mortality, as well as the litter weight at birth and weaning.

**Table 3:** Effect of dietary levels of garden cress seeds on some reproductive parameters of doe rabbits.

Parameters	Garden cress seeds (%)				SEM	P value			
	0	3	4.5	6		Treat	Linear	Quadratic	Cubic
**Litter size, no at:**									
Birth	8.00^c^	11.33^a^	8.50^bc^	9.60^b^	0.36	0.0001	0.0001	0.0001	0.0001
7th day	3.75^b^	8.50^a^	5.75^b^	6.00^ab^	0.56	0.009	0.004	0.014	0.014
14th day	2.75^b^	7.00^a^	4.75^ab^	5.60^a^	0.52	0.018	0.012	0.020	0.020
21st day	2.50	6.50	4.50	4.80	0.55	0.058	0.033	0.065	0.065
28th day	2.25	5.83	3.75	4.80	0.42	0.115	0.679	0.670	0.067
Pre-weaning mortality (0–28 days)	5.75	5.50	4.25	4.80	0.42	0.652	0.722	0.482	0.48
**Litter weight, g. at:**									
Birth	483.75	527.50	498.75	458.00	8.48	0.178	0.106	0.236	0.236
7th day	511.67^b^	801.67^a^	665.00^a^	667.00^a^	31.16	0.005	0.002	0.011	0.011
14th day	571.67^**c**^	1045.00^a^	720.00^bc^	857.00^ab^	52.46	0.003	0.003	0.001	0.001
21st day	750.00^b^	1393.3^a^	958.75^ab^	1126.00^ab^	83.26	0.033	0.015	0.014	0.014
28th day	905.00	1713.30	1187.5	1511.00	127.53	0.147	0.091	0.053	0.053

*SEM* stander error means.

Means with different superscripts in the same row are significantly different (p < 0.05).

**Figure 1:**
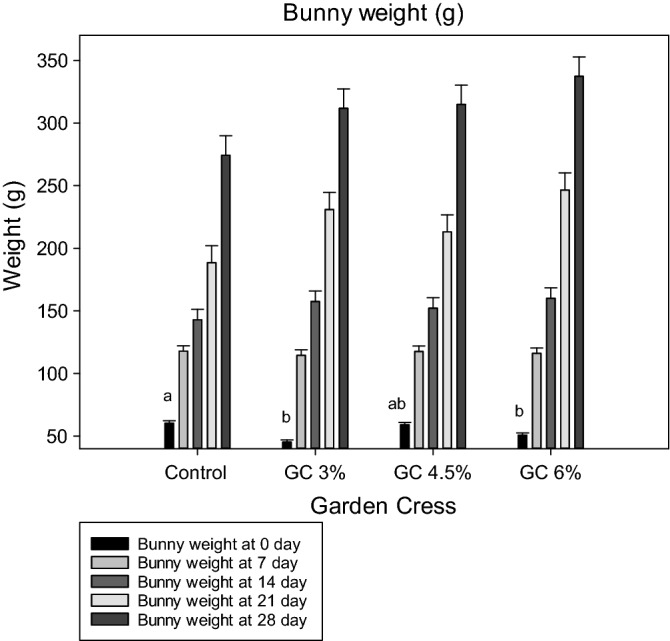
Effect of dietary levels of garden cress seeds on bunny weight of doe rabbits.

Investigations also revealed that the inclusion of GC seeds (*P* < 0.05) increased the litter weight on the 7th day compared with that of the control group. Bunny weights are presented in Fig. 1. Results showed that the bunny weight at birth significantly decreased with GC 3% and 6% treatments compared with that of the control group, with no difference between GC 4% and the control groups, although the total litter weight at birth not effected by GC treatments. However, from the 7th day until weaning, the GC-supplemented group showed similar (*P* > 0.05) bunny weight results compared with that of the control group.

### Milk yield

Results are illustrated in Table 4 showed that the dietary inclusion of GC seeds tended to increase milk yield on the 7 th, 14th and 21st days. However, the milk yield at weaning improved significantly (P = 0.020) by the supplementation of GC seeds at 3 and 6% in doe rabbit diets as compared to the GC 4.5% and control groups.

**Table 4:** Effect of dietary levels of garden cress seeds on milk yield of doe rabbits.

Parameters	Garden cress seeds (%)				SEM	P value			
	0	3	4.5	6		Treatment	Linear	Quadratic	Cubic
**Milk yield (g) at**									
7th day	46.67	49.17	41.25	46.00	4.02	0.125	0.508	0.924	0.924
14th day	105.00	122.5	136.25	117.00	5.65	0.403	0.286	0.563	0.563
21st day	45.00	65.00	50.00	51.00	5.39	0.601	0.415	0.315	0.315
28th day	17.50^b^	40.83^a^	30.00^ab^	40.00^a^	3.15	0.020	0.035	0.027	0.027

*SEM* stander error means.

Means with different superscripts in the same row are significantly different (p < 0.05).

### Haematological parameters

The effects of GC seed supplementation on the haematological parameters of does at weaning are illustrated in Table 5. The different experimental treatments insignificantly affected most haematological parameters, except the Hb concentration, haematological indices (MCH, MCHC), and leukocytes. Specifically, GC seed treatments significantly increased the Hb concentrations in lactating female rabbits compared with that of the control group.. The results also demonstrated that the haematological indices related to Hb, such as MCH and MCHC, considerably increased in the groups of doe rabbits treated with GC seeds (4.5 and 6%) compared to the other groups. Though leukocytes were significantly reduced by GC supplementation, no difference was observed between GC 3% and the control rabbits.

**Table 5:** Effect of dietary levels of garden cress seeds on haematological parameter of doe rabbits.

Parameters	Garden cress seeds (%)				SEM	P value			
	0	3	4.50	6		Treatment	Linear	Quadratic	Cubic
Erythrocytes (10^6^/mm^3^)	5.82	5.77	5.44	5.35	0.11	0.369	0.107	0.928	0.602
Hb (mg/dl)	11.15^c^	12.36^b^	13.24^a^	13.22^a^	0.27	0.0001	0.0001	0.005	0.449
PCV (%)	50.10	50.18	50.20	50.50	0.11	0.996	0.836	0.933	0.954
MCV (fL)	86.31	87.08	92.37	94.72	1.79	0.293	0.077	0.82	0.632
MCH (pg)	19.23^b^	21.51^b^	24.34^a^	24.80^a^	0.77	0.006	0.001	0.321	0.463
MCHC (g/dL)	2.22^b^	2.43^ab^	2.64^a^	2.62^a^	0.06	0.017	0.004	0.129	0.722
Platelets (10^3^/mm^3^)	2.88	2.70	2.71	2.90	7.16	0.463	0.012	1.374	0.002
leukocytes (10^3^/mm^3^)	6.99^a^	6.15^ab^	5.45^b^	5.18^b^	0.24	0.006	0.001	0.319	0.818

*Hb* haemoglobin, *PCV* packed cell volume, *MCV* mean corpuscular volume, *MCH* mean corpuscular haemoglobin, *MCHC* mean corpuscular haemoglobin concentration, *SEM* stander error means.

Means with different superscripts in the same row are significantly different (p < 0.05).

### Blood biochemicals

**Figure 2:**
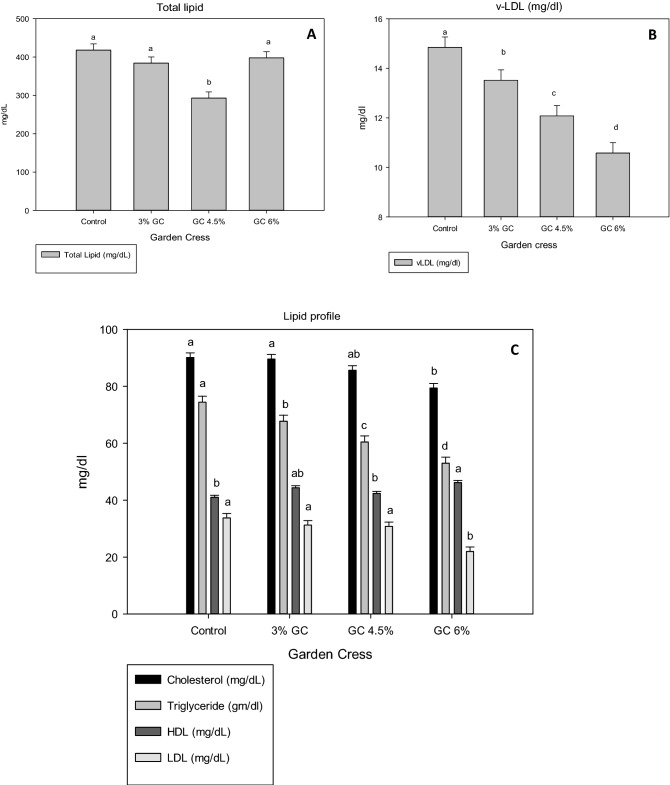
Effect of dietary levels of garden cress seeds on serum lipid profile (**A** total lipid; **B** v-LDL and **C** cholesterol, triglyceride, HDL and LDL) of doe rabbits.

**Figure 3:**
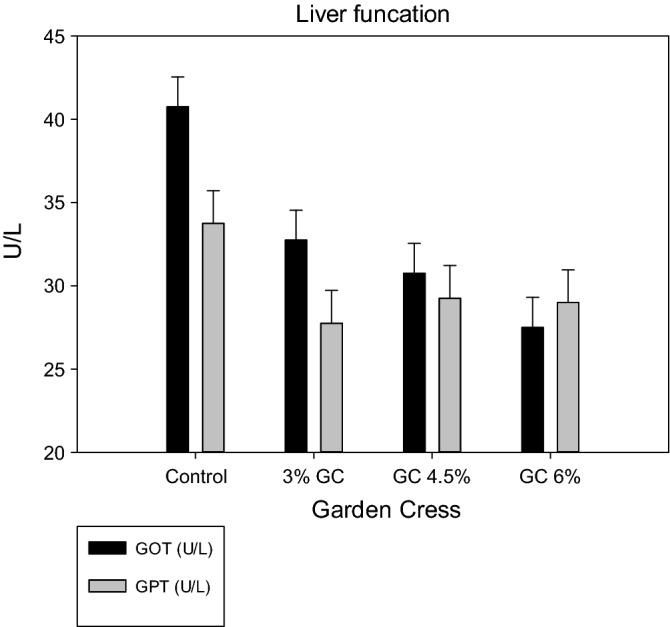
Effect of dietary levels of garden cress seeds on serum liver function of doe rabbits.

The blood lipid profile of does was significantly influenced by GC supplementation (Fig. 2). Investigations showed that the total serum lipid level was significantly decreased in GC 4.5% compared with that of the other groups. However, cholesterol level decreased significantly with GC 6% and insignificantly with GC 4.5% compared to that in the GC 3% and control groups. However, all GC treatments succeeded in lowering the serum TG and v-LDL concentrations compared with those of the control group. The dietary supplenetation of GC seeds at the highest level (6%) significantly declined LDL and significantly increased HDL compared to other groups. Figure 3 shows that liver functions did not affect in all experimental groups, as reflected by the estimated values of serum GOT and GPT.

**Figure 4:**
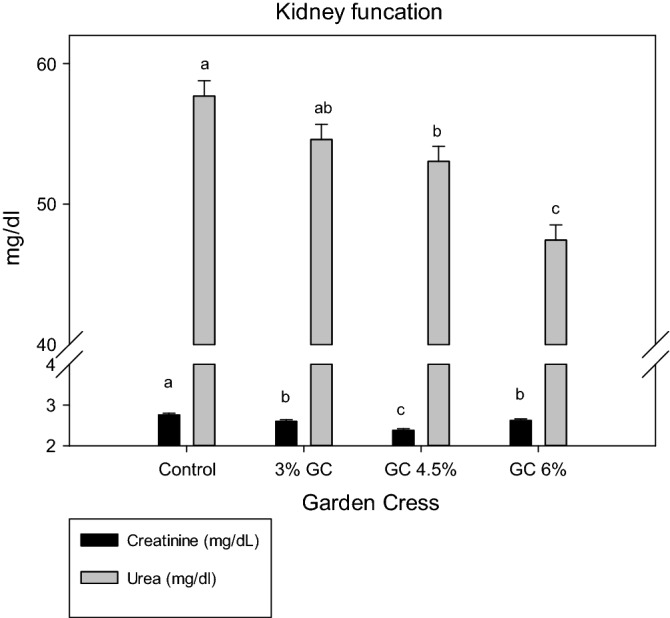
Effect of dietary levels of garden cress seeds on serum kidney function of doe rabbits.

Regarding kidney function (Fig. 4), the inclusion of GC seeds significantly decreased the serum creatinine and urea levels in the does compared with that of the control group, with a nonsignificant difference between the GC 3% and control groups for the urea levels. The TAC increased significantly by supplementation with only GC 6%, SOD improved considerably by supplementation with GC seeds at different concentration compared with the control group (Table 6).

**Table 6:** Effect of dietary levels of garden cress seeds on serum antioxidants profile of doe rabbits.

Parameters	Garden cress seeds (%)				SEM	P value			
	0	3	4.50	6		Treatment	Linear	Quadratic	Cubic
TAC (mmol/l)	1.48^b^	1.66^b^	1.64^b^	2.02^a^	0.07	0.014	0.003	0.039	0.076
SOD (u/ml)	1.45^c^	1.60^b^	1.75^a^	1.82^a^	0.04	0.0001	0.0001	0.397	0.701

Means with different superscripts in the same row are significantly different (p < 0.05).

*SEM* stander error means.

## Discussion

Garden-cress (Lepidium sativum) seeds has been considered as a very useful medicinal plant and contain many nutrients and phytochemicals with a wide range of important biological functions^4^. The absence of a significant variation in the does’ body weights through mating, palpation, and parturition in all experimental treatments indicated no negative effect of the dietary supplementation of GC seeds. However, results showed a numerical improvement in the conception rate or gestation period due to the inclusion of CG seeds in the doe diets. The current results agree with those of Imade^19^**,** who observed that gestation lengths following supplementation with 5–10% GC seed were insignificant, with a dose-dependent significant increase (*P* < 0.05) in the conception rate of 5–7% GC seeds.

Additionally, results showed that although 3% GC treatment achieved the best results in litter weight on the 7th, 14th and 21 st days, it achieved the highest litter size at birth, on the 7th and 14th days. However, the control rabbits recorded the highest bunny weight at birth due to the low litter size of the control group. In disagreement with this study, Imade^19^ reported that litter weights and the number of live kits born significantly (*P* < 0.05) decreased in all GC seed groups (5%, 7%, and 10%), and this difference in results was proposed to be due to the GC seed doses used in that study.

The positive effect of GC seed addition on most reproductive parameters is due to the phytochemicals in GC seeds, which enhance the reproductive hormones and antioxidant status^20^. In a previous study, they observed that the phytochemicals in GC seeds significantly increased the hypothalamic–pituitary–gonadal axis activity, which boosted serum luteinizing hormone (LH) and follicle-stimulating hormone (FSH) concentrations^21^. Similarly, in another research, GC seeds raised the levels of estrogen, progesterone, LH, FSH, and free testosterone hormones in does^22^, which was attributed to the phytosterol^23^ and phytoestrogen^19^ components of GC seeds. These ingredients were associated with a temporary or permanent modification of the feedback loop between the hypothalamus, pituitary, and gonad glands, which influenced the responses of endogenous estrogen^19^.

Furthermore, investigations showed that although the effect of GC seeds on the milk yield of female rabbits did not appear throughout the first three weeks of lactation, the milk yield significantly increased in the last week of lactation before weaning (28th day), especially in the GC 3% and 6% groups. These results agree with those of Singh and Paswan^24^ in humans, Falana et al.^25^ in sheep, and Abo El-Nor et al.^26^, and Kumar et al.^27^ in buffaloes. These authors observed that consuming GC seeds after parturition increased total milk yield compared with the control group. These results are proposed to be due to the pharmacological effects of GC seeds through interaction with dopamine receptors, resulting in increased prolactin levels, thereby augmenting milk production^28^. This increase in milk yield could also be associated with the antioxidant components^29,30^ of GC seeds, such as phenols and tannins.

Generally, it has been reported that the values of haematological parameters of healthy rabbits fall within the safety limit^31^. Therefore, normal haematological values reveal the physiological status of does. This study showed that GC seed treatments positively influenced the Hb levels and haematological indices related to Hb, such as MCH and MCHC, with an opposite effect on leukocyte count. The improvement in Hb, MCH, and MCHC suggests that GC seeds enhanced nutrient use and absorption into the bloodstream of lactated rabbits, improving blood formation due to the availability of essential nutrients and mineral elements, especially iron, manganese, copper, and calcium in GC seeds^32^. In agreement with this research, Falana^25^, including Singh and Paswan^24^, also reported that GC seeds boosted the blood Hb concentrations. Specifically, these authors explained that the high contents of folic acids, iron, and vitamin C in GC seeds accounted for the standard Hb levels, thereby increasing blood counts. A study also showed that the dietary addition of GC seeds to does’ diets decreased the leukocyte count, indicating that it improved leukocyte function and has anti-inflammatory activity^33^. However, Mahassni and Khudauardi^4^ disagreed with these results, showing that GC seeds significantly increased the mean leukocyte count compared with that of the control group.

Additionally, our results showed that GC supplementation significantly improved the serum lipid profile of does supplemented with GC seeds. Similar results were documented by Chauhan et al.^34^, Korish and Arafah^35^, and Althnaian^7^, who revealed that GC seeds improved blood lipids by reducing total serum cholesterol and TG levels. Yousef et al.^8^ also reported a significant decrease in LDL-c and v-LDL-c levels using GC seed oil compared with the control group. Hyperlipidemia could be due to the enhanced release of abundant free fatty acids and decreased lipoproteins^7^ by inhibiting cholesterol biosynthesis or β-hydroxy β-methylglutaryl-CoA reductase (the rate-limiting enzyme that mediates the first step in cholesterol biosynthesis). Therefore, biochemical changes and enzyme activity alterations induced by stress on liver function or reduced hepatic synthesis of fatty acids could decrease the TG concentration^8^ or reduce the absorption of lipids, enhancing their excretion^34^.

This study also showed that a decrease in the serum levels of cholesterol, LDL, v-LDL, and TG, including an increase in HDL level in the blood of does receiving GC seeds, was probably due to the presence of glycosides, alkaloids, tannins (phenolic compounds), flavonoids^1^, and amino acids (glutamine, cysteine, glycine). These active components in GC seeds may also have antioxidant activities, resulting in endogenous antioxidant glutathione synthesis^36^ or caffeic acid-based antioxidant effects^37^. Furthermore, these antioxidant effects may be due to the inhibitory effect of GC on the reactive generation of oxygen species and an increase in the mitochondrial membrane potential^38^. Therefore, the results in this study proved this previous effect by the high serum antioxidant (TAC and SOD) levels observed in the investigated rabbits. The hyperlipidemic properties of GC seeds could also be due to the high copper content of the GC seeds^32^, which has blood cholesterol-lowering effects.

Serum liver functions (GPT and GOT) are closely correlated with most cases of liver disorder. However, liver functions were undisturbed in all experimental groups in this study, which agreed with that of Ghada et al.^8^ and Shivangi et al.^39^. The results on liver function in this study disagreed with those of Abdella et al.^20^, who revealed that rabbits treated with oils and GC seed extracts had significantly decreased concentrations of blood GPT and GOT compared with those of the control group. The authors also observed that treating rabbits injured with carbon tetrachloride for 5 and 10 weeks with GC seeds significantly repaired their liver enzymes^40,41^. Alternatively, the positive effects of GC seeds on serum creatinine and urea of does observed in thus study reflected kidney function improvement. In agreement with this finding, Al Hamedan^42^ demonstrated that GC seeds suppressed urea and creatinine levels, reducing the risk of acute kidney failure in rats^9,10^. This improvement in renal function was due to the active compounds in GC seeds, such as flavonoids and steroidal compounds, as demonstrated in this study, which increase glomerular filtration rate^43^. Moreover, GC seed also has nephroprotective and curative activities^42^. A previous study reported the ability of GC seeds' aqueous extract to improve the kidneys and restore electrolyte balance and renal functions in sodium nitrite-treated rats^43^. However, flavonoids and phenolic compounds of GC could protect against diabetic nephropathy in streptozotocin-induced diabetic rats, which improved blood urea nitrogen, creatinine, and urine extraction and kidney tissue damage, with a reduction in mitochondrial damage^44^. Therefore, given that there is a sense of balance between the production and neutralization of ROS in the biological body system, this balance is maintained by the presence of natural antioxidants^45^ such as TAC and SOD. However, under pregnancy and lactation stress, this balance may be disturbed^46^.

## Conclusion

Dietary supplementation with different levels of GC seeds in the rabbit diets could be considered a positive method for enhancing the reproductive performance and milk yield of does. Moreover, the inclusion of GC seeds in the diet of rabbits can improve their lipid profiles, kidney function, and antioxidant status. Based on these results, it is recommended that the supplementation of GC seeds in the diet was effective in improving the reproductive performance, milk yield, blood lipid profile, and antioxidant status of does.

## Data Availability

The data used to support our study’s findings are included in the article, and data coding is available from the corresponding author upon reasonable request.

## Abbreviations

GC: Garden cress
HDL: High-density lipoprotein
LDL: Low-density lipoprotein
vLDL: Very-low-density lipoprotein
PCV: Packed cell volume
Hb: Hemoglobin concentration
PLT: Platelets
MCV: Mean cell volume
MCH: Mean cell hemoglobin
MCHC: Mean cell hemoglobin concentration
GOT: Glutamic-oxaloacetic transaminase
GPT: Glutamic pyruvic transaminase
TAC: Total antioxidant capacity
SOD: Superoxide dismutase
ANOVA: Analysis of variance
ROS: Reactive oxygen species
